# The emerging role and therapeutic targeting of autophagy-lysosome pathway in the pathogenesis of Parkinson’s disease

**DOI:** 10.1186/s40035-026-00555-3

**Published:** 2026-05-07

**Authors:** Takahiro Shimizu, Sanem Isik, Nitika Kamath, Zhenyu Yue

**Affiliations:** Department of Neurology and Neuroscience, Center for Parkinson’s Disease Neurobiology, Icahn School of Medicine at Mount Sinai, New York, NY 10029 USA

**Keywords:** Parkinson’s disease, Autophagy-lysosome pathway, Lysosomal homeostasis, α-Synuclein, Secretory autophagy

## Abstract

Parkinson’s disease (PD) is a progressive neurodegenerative disorder characterized by dopaminergic neuron loss and the accumulation of misfolded α-synuclein, yet the underlying mechanisms remain incompletely understood. Over the past two decades, genetic discoveries have highlighted the convergence of multiple familial PD genes on the autophagy-lysosome pathway (ALP), a key cellular system responsible for the degradation and recycling of intracellular components. Recent studies have further revealed that components of the ALP not only mediate the clearance of α-synuclein aggregates but also, under certain pathological conditions, contribute to their propagation via lysosomal exocytosis or secretory autophagy. The precise functions of autophagy are highly context-dependent, with neuronal and glial cells exhibiting distinct ALP dynamics that shift with development, stress, and aging. In this review, we summarize current knowledge on the physiological regulation of autophagy in the brain and critically examine its involvement in PD pathogenesis, incorporating mechanistic insights from familial models and emerging evidence from sporadic PD. We also explore translational implications, focusing on efforts to identify ALP-related biomarkers in cerebrospinal fluid and urine, and on the therapeutic potential of modulating ALP activity. Although the causality between ALP dysfunction and PD remains elusive, mounting evidence supports its contribution to disease progression, particularly through impaired lysosomal homeostasis and disrupted intracellular trafficking. Future research should aim to define cell type-specific ALP alterations, clarify the bidirectional interactions between α-synuclein and autophagic machinery, and develop in vivo tools to monitor autophagy activity and secretory signatures. A deeper understanding of these processes will be crucial for refining PD models, discovering robust fluid biomarkers, and designing targeted therapies capable of modifying disease trajectory.

## Background

Parkinson’s disease (PD) is the second most common neurodegenerative disorder worldwide [1]. PD is characterized by progressive motor symptoms such as bradykinesia along with the progressive loss of dopaminergic neurons in the substantia nigra and the accumulation of aggregated α-synuclein in Lewy bodies and Lewy neurites [1]. PD prevalence increases with aging, and its burden is expected to grow with the aging population [1]. Despite decades of research, the pathogenic mechanisms underlying PD remain incompletely understood, and disease-modifying therapy is still unavailable.

Genetic studies have provided crucial insights into the molecular underpinnings of PD. Mutations in genes such as synuclein alpha (*SNCA*), Parkin RBR E3 ubiquitin protein ligase (*PRKN*), PTEN-induced putative kinase 1 (*PINK1*), Parkinsonism-associated deglycase (*Park7*; encoding DJ-1), leucine-rich repeat kinase 2 (*LRRK2*), vacuolar protein sorting 35 (*VPS35*), and vacuolar protein sorting 13C (*VPS13C*) have been identified as causal for familial PD, whereas mutations in glucosylceramidase beta 1 (*GBA1*) represent major genetic risk factors for PD [2, 3]. Many of these genes are functionally related to the autophagy–lysosome pathway (ALP). Remarkably, genome-wide association studies (GWAS) have revealed multiple ALP-related loci as risk factors for sporadic PD (sPD), suggesting that ALP dysfunction is not confined to familial cases but may be a common denominator across the disease spectrum [4]. These findings prompted the somewhat oversimplified notion that the ALP impairment may be a major driver of PD pathogenesis.

Autophagy is a highly conserved intracellular degradation system found in most eukaryotic organisms that delivers cytoplasmic substrates to lysosomes [3, 5]. Advances in molecular biology have delineated the complex machinery that governs autophagosome formation, cargo selectivity, and subsequent cargo degradation. Meanwhile, human genetics has uncovered diseases caused by mutations in core autophagy-related (*ATG*) genes [6, 7]. Interestingly, the presence of long-term survivors carrying such mutations suggests that the impact of autophagy on cellular and organismal health is not uniformly essential through life, but may vary across developmental stages and aging. This implies that the physiological function of autophagy is context-dependent and dynamically modulated over the lifespan, especially in the brain. Importantly, α-synuclein has emerged as a substrate of autophagy [8–10]; however, it may also act as a potential disruptor and even utilize the autophagic machinery for its own propagation under certain conditions [11–14]. This raises the possibility that the ALP, while generally protective, might in some contexts contribute to the spread of pathology. Thus, the relationship between autophagy and PD pathogenesis is increasingly recognized as multifaceted, involving not only protective but also, in certain contexts, destructive, contributing to disease progression.

In this review, we summarize recent advances in understanding how autophagy intersects with pathogenic pathways of PD, focusing on genetic evidence, mechanistic insights, and emerging translational implications. We aim to provide a framework for future studies that may clarify whether targeting the autophagy–lysosome system can ultimately translate into effective disease-modifying therapies.

## The cellular processes and functions of ALP in brain

### General architecture of autophagy and neuron-specific regulations

Autophagy is categorized into three distinct types: macroautophagy, microautophagy, and chaperone-mediated autophagy (CMA) [5]. In macroautophagy, a double-membraned isolation membrane elongates to engulf cytoplasmic contents, forming an autophagosome that subsequently fuses with a lysosome for degradation, whereas cytosolic components are directly taken up through invagination of the lysosomal membrane in microautophagy [5]. In CMA, substrate proteins harboring a KFERQ-like motif are selectively recognized by heat shock cognate 71-kDa protein (HSC70) and directly translocated into lysosomes through multimerized lysosome-associated membrane protein type 2A (LAMP2A), without involving membrane remodeling [15]. Among these, macroautophagy has been the most intensively studied, both mechanistically and functionally. In contrast, the physiological functions of microautophagy and CMA remain incompletely understood [5]. Accordingly, this review places primary emphasis on macroautophagy. Nonetheless, CMA is specifically discussed within the framework of PD pathophysiology, given its emerging significance in disease-related mechanisms.

**Fig. 1:**
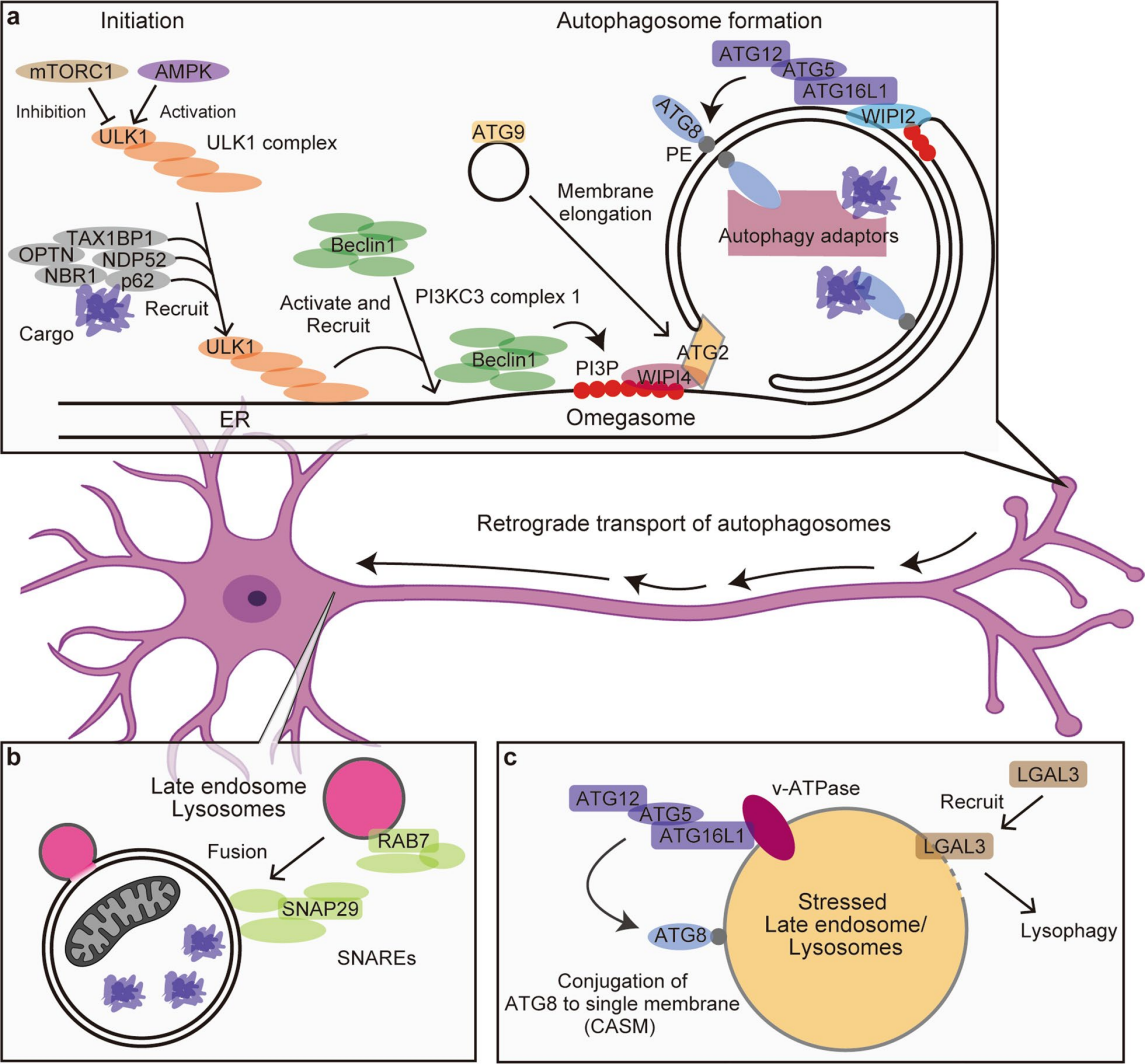
Overview of autophagy initiation, autophagosome maturation, and lysosomal repair mechanisms. **a** The initiation of autophagy includes activation of the ULK1 complex, which is negatively regulated by mechanistic target of rapamycin complex 1 (mTORC1) and positively regulated by AMP-activated protein kinase (AMPK). Autophagy adaptors (receptors) can facilitate the recruitment of the ULK1 complex. The PI3KC3 complex 1 (containing Beclin1/Atg14L) generates PI3P-enriched membranes on the endoplasmic reticulum (ER), which recruit WIPI2 and WIPI4. These factors promote the recruitment of the ATG8 conjugation machinery and the lipid transfer protein ATG2 respectively. Autophagic substrates are captured directly by ATG8-family proteins or via autophagy adaptors and are sequestered into the autophagosome. **b** After formation, autophagosomes undergo retrograde transport toward the perinuclear region, during which they gradually acidify. Fusion with lysosomes is mediated by SNARE proteins such as SNAP29. **c** Under lysosomal overload stress, ATG16L1 is recruited to the lysosomal membrane via v-ATPase, initiating conjugation of ATG8 to single membranes (CASM). In cases of membrane integrity loss, galectin-3 is recruited to damaged lysosomes initiating lysophagy

During macroautophagy (hereafter referred to as “autophagy” unless otherwise specified), proteins encoded by *ATG* genes coordinate autophagosome biogenesis [5]. The process is initiated by the Unc51-like kinase (ULK) complex [16], which activates class III PI3K (phosphatidylinositol 3-kinase) (Fig. 1a) [17]. This generates phosphatidylinositol 3-phosphate (PI3P) on the endoplasmic reticulum (ER) or isolation membrane, where autophagosome formation starts (Fig. 1a) [17]. PI3P recruits WIPI2 and WIPI4, which in turn recruit downstream components such as ATG16L1 and ATG2, respectively (Fig. 1a) [18, 19]. ATG2 transfers lipids for membrane elongation [20], while ATG16L1 forms a complex with the ATG12–ATG5 conjugate to lipidate ATG8 family proteins, comprising LC3 (microtubule-associated protein light chain 3) and GABARAP (gamma-aminobutyric acid receptor-associated protein) subfamilies in mammals, onto phosphatidylethanolamine on the isolation membrane (Fig. 1a) [21, 22]. This ATG8 conjugation system supports multiple subsequent steps of the autophagy process, including autophagosome formation and lysosomal fusion [5]. Lysosomal fusion critically depends on the coordinated action of soluble *N*-ethylmaleimide-sensitive factor attachment protein receptor (SNARE) proteins, including SNAP29 (Fig. 1b) [5].

Importantly, ATG8 family proteins also mediate substrate selectivity, either directly or indirectly via adaptor or receptor proteins such as p62/Sequestosome 1 (Fig. 1a) [23]. This gives rise to selective forms of autophagy including aggrephagy for protein aggregates and mitophagy for mitochondria, each involving distinct adaptor proteins [23]. Notably, adaptor proteins can also induce autophagy by helping recruit the ULK1 complex, extending their function from selectivity to initiation (Fig. 1a) [23]. In addition, autophagy is not limited to degradation; when lysosomal fusion is impaired, cargo may be secreted via a process known as secretory autophagy [24, 25]. In recent years, it has become increasingly evident that *ATG* genes also participate in non-canonical functions [5]. For instance, ATG8 can be conjugated to single membranes of endolysosomal compartments, in a process termed conjugation of ATG8 to endolysosomal single membranes (CASM), which involves several core autophagy machinery components (Fig. 1c) [26]. CASM can be activated by various lysosomal stresses including lysosomal overload. In contrast, lysosomal membrane rupture is recognized by galectin-3 and related sensors, triggering lysophagy, a selective autophagy pathway that removes damaged lysosomes (Fig. 1c) [27, 28]. In parallel, CASM has also been implicated in maintaining lysosomal homeostasis, although its physiological significance is only beginning to be understood.

One of the critical roles of autophagy is the degradation of unwanted cytoplasmic components to maintain cellular homeostasis [3, 5]. This function is especially vital in postmitotic cells such as neurons [3]. Indeed, conditional knockout (KO) of *Atg5* or *Atg7* in neurons results in neurodegeneration in central nervous system (CNS), accompanied by intracellular protein aggregates [29, 30]. Particularly, deletion of *Atg7* gene specifically in midbrain dopaminergic neurons causes progressive axonal and dendritic dystrophy, followed by the loss of soma and motor function deficits in aged mice [31]. Although the molecular machinery is largely conserved, neurons require spatially specialized regulation due to their highly polarized and extended structure [3, 32]. Namely, autophagosomes are primarily formed at distal axons and undergo retrograde transport toward the soma for degradation [33]. In addition to such spatial regulation of autophagosome formation, autophagic substrates in neurons are highly diverse and may differ even across neuronal subtypes [34–36]. Moreover, autophagy substrates also appear to change during development, indicating dynamic shifts in its functional relevance throughout neuronal maturation [37].

Autophagy is a constitutive degradation process that controls neuronal homeostasis in the soma, axon, and dendrites [31, 33], while activity- or stress-dependent regulation of autophagy has also been described in presynaptic terminals and dendrites. Notably, several autophagy-related proteins have been detected in close proximity to synaptic vesicles [38], and in *C. elegans*, presynaptic autophagosome formation via ATG9 has been suggested to be triggered by the activity-dependent exo-endocytic cycling of synaptic vesicle membranes [39]. This “synaptic autophagy” may contribute to the regulation of neurotransmitter release, synaptic plasticity, and extracellular signaling [32]. Given the high abundance of α-synuclein at synaptic terminals [40, 41], perturbations in synaptic autophagy may underlie early pathogenic events in neurodegeneration. Consistent with this notion, the presynaptic trafficking protein synaptojanin-1 has been shown to regulate autophagy and α-synuclein homeostasis at presynaptic terminals, as synaptojanin-1 haploinsufficiency in mice leads to detergent-insoluble p62/LC3 accumulation together with increased α-synuclein and degeneration of dopaminergic terminals [42].

### Glial autophagy

Autophagy in glial cells has also gained increasing attention in recent years. Like neurons, glial cells may utilize autophagy in a context-dependent manner. Astrocytes, microglia, and oligodendrocytes appear to process distinct substrates, reflecting their specialized functions [43]. In the case of astrocytes, one of their key roles is lipid handling within the CNS, partially through metabolic coupling with neurons [44, 45]. Under conditions of elevated neuronal activity or oxidative stress, neurons preferentially synthesize fatty acids but export excess or potentially toxic lipids to neighboring astrocytes, where they are sequestered as lipid droplets [44, 45]. Given that lipid droplets can be degraded through autophagy, a process referred to as lipophagy [46], lipophagy in astrocytes may be particularly important [47]. Notably, astrocytic lipid handling relies not only on lipid droplet sequestration but also on mitochondrial metabolism to detoxify excess fatty acids [44]. This process may impose a substantial mitochondrial burden on astrocytes. Importantly, however, astrocytes are thought to possess a high mitophagic capacity [48], a feature that may not only support mitochondrial quality control within astrocytes but may also indirectly contribute to limiting the toxicity of neuron-derived fatty acids, thereby helping to maintain the CNS-wide homeostasis.

Microglial autophagy operates on a wide range of substrates and fulfills distinct functions across developmental and pathological contexts. During development, for example, microglial autophagy is essential for synaptic pruning, a process required for the refinement of neural circuits [49, 50]. In the adult brain, microglia-specific deletion of *Atg5* in mice leads to age-dependent inflammasome activation and increased production of proinflammatory cytokines under basal conditions [51]. This finding indicates that, in microglia as the resident innate immune cells of the CNS, autophagy-related LC3 lipidation pathways contribute to restraining inflammasome-associated signaling, thereby preventing excessive or inappropriate inflammatory activation in the adult brain [51]. Consistent with this notion, a study using primary microglia with *Atg7* deletion has revealed prominent disturbances in lipid homeostasis accompanied by elevated inflammatory responses, including lipid droplet accumulation and impaired fatty acid utilization [52]. Similar lipid-associated abnormalities and microglial activation have also been observed in microglia isolated from *Atg7* conditional KO mice, indicating that Atg7-dependent autophagy pathways are required to restrain lipid-driven inflammatory stress in microglia [52].

In neurodegenerative contexts, selective autophagy in microglia has been shown to directly mediate the clearance of pathogenic protein substrates [10]. Neuron-released α-synuclein is efficiently taken up by microglia and delivered to double-membraned autophagosomes for lysosomal degradation, providing direct evidence that autophagy participates in the removal of extracellularly derived pathogenic proteins [10]. Disruption of this autophagic process leads to increased neuronal α-synuclein accumulation and accelerates neurodegeneration, underscoring a neuroprotective role of microglial macroautophagy in disease-relevant settings [10].

### Autophagy during aging

Autophagic activity has long been considered to decline with aging across diverse organisms [53], and this notion prompts the hypothesis that reduced autophagic capacity may contribute to the pathogenesis of age-related disorders, including PD, in which aging represents a major risk factor [54, 55]. Consistent with this view, age-associated attenuation of autophagy has been supported by multiple observations, including reduced autophagic vesicle abundance in the aged mouse cerebral cortex [56] as well as transcriptional downregulation of core autophagy-related genes such as *ATG5*, *ATG7*, and *BECN1* in aged human cortical tissue [57]. However, the extent to which findings from non-human models can be directly extrapolated to human aging remains uncertain, and it is also unclear how age-dependent changes in the expression or abundance of autophagy-related proteins translate into alterations in dynamic autophagic flux [53].

Recent in vivo studies using quantitative reporters of autophagic flux have begun to reveal a more complex picture, in which age-related changes in autophagy are not uniformly suppressive but instead vary across cell types, brain regions, and selective autophagy pathways [53]. Studies employing quantitative in vivo reporters of autophagic flux have further refined this view, demonstrating that age-associated alterations in autophagy are highly region-dependent within the brain. In these analyses, autophagic activity is not uniformly reduced across the entire brain, but instead exhibits region-specific patterns characterized by decline in some areas while remaining relatively preserved in others [58]. Moreover, mitophagy does not appear to undergo a global age-dependent decline; rather, mitochondrial turnover is maintained or even increased in a region- and cell type-dependent manner during normal aging, indicating that the trajectories of selective autophagy pathways are intrinsically heterogeneous [58]. Importantly, autophagy can be functionally divided into the basal constitutive activity and the inducible activity in response to stress, and these two are differentially affected by aging. While basal autophagy may be maintained or upregulated in aged tissues to cope with chronic cellular stress, the capacity to further enhance autophagic flux in response to acute metabolic or environmental stress is often blunted with age, as demonstrated in a genetically tractable mammalian model [59]. Taken together, these findings underscore that the age-related changes in autophagy cannot be adequately captured by single molecular markers or limited flux measurements, and that simplistic classification of autophagy as either “increased” or “decreased” with aging should be avoided.

Moreover, beyond age-associated changes in autophagic activity per se, the impact of autophagy on the aging process appears to be context-dependent. For example, in microglia, autophagy has been shown to suppress cellular senescence and support microglial responses to amyloid pathology, thereby facilitating plaque-associated clearance mechanisms in Alzheimer’s disease model mice [60]. Conversely, there are settings in which age-associated enhancement of autophagy contributes to disease pathogenesis. In adipose tissue, excessive autophagic activity driven by age-dependent loss of the autophagy inhibitor Rubicon promotes metabolic dysfunction, highlighting that increased autophagy can be maladaptive in specific cell types during aging [61]. In addition, autophagy-mediated degradation of specific nuclear substrates, such as WSTF, has been shown to act as a molecular switch for chronic inflammatory activation in aged cells, illustrating that selective cargo turnover by autophagy can directly precipitate pathological states [62].

When considering the pathological relevance of age-associated alterations in autophagy, insights can also be drawn from patients harboring mutations in core *ATG* genes. Such patients frequently exhibit neurodevelopmental abnormalities, underscoring the indispensable role of autophagy in brain development [6, 7]. However, because severe impairment of autophagy is often incompatible with survival in animal models, it remains unclear how autophagy dysfunction directly causes neurodegenerative disease in adults. Moreover, the physiological roles of autophagy vary across cell types and developmental stages, further complicating efforts to disentangle the pathogenic mechanism. In this regard, insights from familial PD, in which autophagy-related pathways are frequently implicated, may offer valuable perspectives in understanding the broader link between autophagy and neurodegeneration.

## ALP in PD

### Overview

ALP plays a critical role in controlling the homeostasis of α-synuclein, which accumulates in intraneuronal inclusions in PD [63]. Investigations of causative genes of PD revealed a convergence of ALP dysfunction underlying pathogenic pathways of PD. The clinical and pathological features of established familial PD genes are summarized (Table 1). Among them, specific genes with well-documented link to ALP are discussed here. For example, *PRKN/PINK1* and *VPS13C* have been implicated in the autophagy machinery. *LRRK2* is activated and localized to stressed lysosomes to repair injured lysosomes. *GBA1* is a major genetic risk factor of PD and encodes a lysosomal enzyme. Finally, we review emerging evidence of autophagy impairment in sPD.

**Table 1:** Monogenic PD genes with clinicopathological and genetic characteristics

Gene name	Inheritance	Clinical characteristics	Lewy pathology	GWAS-sPD link	References
*SNCA*	AD	Early onset (mutation or triplication), Late onset (duplication)	(+)	Yes	[64–69]
*PRKN*	AR	Early onset, slow progression	Rare	No	[70–72]
*PINK1*	AR	Early onset, slow progression	Rare	No	[72, 73]
*PARK7*	AR	Early onset, atypical parkinsonism	(+)	No	[74]
*LRRK2*	AD	Almost identical to sPD	70%–80%	Yes	[2, 4, 72, 75–79]
*ATP13A2*	AR	Early onset, atypical parkinsonism	(−)**	No	[80]
*PLA2G6*	AR	Early onset, atypical parkinsonism	(+)	No	[81, 82]
*SYNJ1*	AR	Early onset, atypical parkinsonism	(−)**	No	[83, 84]
*POLG*	AD or AR*	Early onset, atypical parkinsonism	(−)**	No	[85, 86]
*VPS35*	AD	identical to sPD	Unknown	No	[87, 88]
*VPS13C*	AR	Early onset, faster progression	(+)	Yes	[4, 89]

*GWAS* genome-wide association study, *sPD* sporadic Parkinson’s disease, *AD* autosomal dominant, *AR* autosomal recessive

^*^Both AD and AR inheritance patterns have been reported in different families carrying variants in this gene

^**^Only a limited number of autopsied cases have been reported

### α-Synuclein and autophagy

α-Synuclein is a presynaptic protein encoded by the *SNCA* gene, which regulates synaptic transmission by promoting SNARE complex assembly and modulating synaptic vesicle trafficking [90]. Under pathological conditions, however, α-synuclein undergoes conformational changes that drive its aggregation. This process is initiated by the misfolding of a central hydrophobic domain within the protein, leading to the formation of β-sheet rich structures that act as self-propagating templates [91]. These misfolded species seed the conversion of native α-synuclein into soluble oligomers and, eventually, into insoluble fibrils, contributing to the hierarchical buildup of Lewy pathology [92]. Importantly, mutations and multiplications in *SNCA* cause autosomal-dominant forms of familiar PD [64, 67–69]. Missense mutations such as A53T, as well as gene triplications, are typically associated with early-onset PD [68, 69, 93], whereas duplications tend to result in later-onset forms that more closely resemble sPD in their clinical course [64, 67]. These distinct genetic alterations affect α-synuclein homeostasis through different mechanisms: point mutations increase the intrinsic aggregation propensity of the protein [93], while multiplications raise its expression levels, thereby increasing the likelihood of spontaneous aggregation [69]. Notably, *SNCA* triplications are associated with earlier onset and more severe phenotypes compared to duplications, indicating a dose-dependent effect [64, 67, 69]. In addition, variants at the *SNCA* locus have been associated with increased risk for sPD in GWAS [65, 66]. These clinical and mechanistic observations provide a strong link between α-synuclein dysregulation and the pathogenesis of PD.

α-Synuclein is degraded through multiple proteolytic pathways, including autophagy, CMA, and the ubiquitin–proteasome system (UPS), as demonstrated in cultured cells [8, 9]. In CMA, the presence of a KFERQ-like motif (VKKDQ) within wild-type α-synuclein is a key determinant for lysosomal targeting, whereas disease-associated alterations, including pathogenic mutations such as A53T and A30P as well as post-translational modifications observed in PD, bind LAMP2A but fail to translocate into the lysosomal lumen, thereby occupying the receptor and interfering with LAMP2A-mediated CMA execution [94, 95]. Importantly, CMA inhibition by α-synuclein not only promotes its own accumulation but also compromises the degradation of other CMA substrates. In particular, impaired CMA-mediated turnover of the neuronal survival factor MEF2D has been reported in α-synuclein-overexpressing models and in the brains of patients with PD, highlighting a pathogenic consequence of CMA dysfunction beyond α-synuclein itself [96]. Moreover, fibrillar forms of α-synuclein impair CMA, UPS as well as ALP [97–99]. Although the reports of intracellular accumulation of α-synuclein and formation of Lewy body-like inclusions in autophagy-deficient neurons support a potential role of autophagy in digesting large α-synuclein aggregates [31, 36, 100], the direct evidence of autophagy clearance of fibril through autophagy is lacking. Notably, microglia clear soluble α-synuclein through autophagy [10]. In PD mouse models expressing human α-synuclein in neurons, microglia take up neuron-derived α-synuclein and degrade it through a selective autophagy process known as synucleinphagy, limiting disease progression [10]. These observations suggest that autophagy plays a key role in suppressing toxic α-synuclein aggregation, at least in mouse models. However, the structure and pathogenic properties of α-synuclein aggregates in the brains of patients may differ from those observed in experimental model systems and such properties may also vary even between PD and dementia with Lewy bodies, both of which develop neuronal α-synuclein accumulation [101]. This uncertainty limits straightforward extrapolation from cellular and animal models to human disease. Furthermore, the mechanisms by which large α-synuclein assemblies, such as fibrils, interact with the autophagy machinery and how they evade autophagic degradation remain incompletely understood.

A major route by which cells eliminate misfolded proteins is aggrephagy, a form of selective autophagy that targets ubiquitinated protein aggregates for degradation [23]. α-Synuclein aggregates are known to be ubiquitinated, enabling their recognition by autophagy adaptors such as p62 and subsequent delivery to autophagosomes [102–104]. An additional pathway is likely to involve direct interaction between α-synuclein fibrils and ATG8 family protein. A recent structural study has demonstrated that LC3B can directly bind to α-synuclein fibrils in vitro, with a stronger affinity than for monomeric α-synuclein [14]. In parallel, experimental evidence has shown that fibrillar α-synuclein is internalized during cell-to-cell transmission and accumulates in lysosomes, where it causes lysosomal membrane damage [28]. Such damage is known to trigger lysophagy, a form of selective autophagy that eliminates damaged lysosomes [28]. While the above evidence does not support a role of autophagy in directly degrading fibrillar α-synuclein, it suggests a protective function of autophagy in removing damaged organelles caused by fibrillar α-synuclein.

**Fig. 2:**
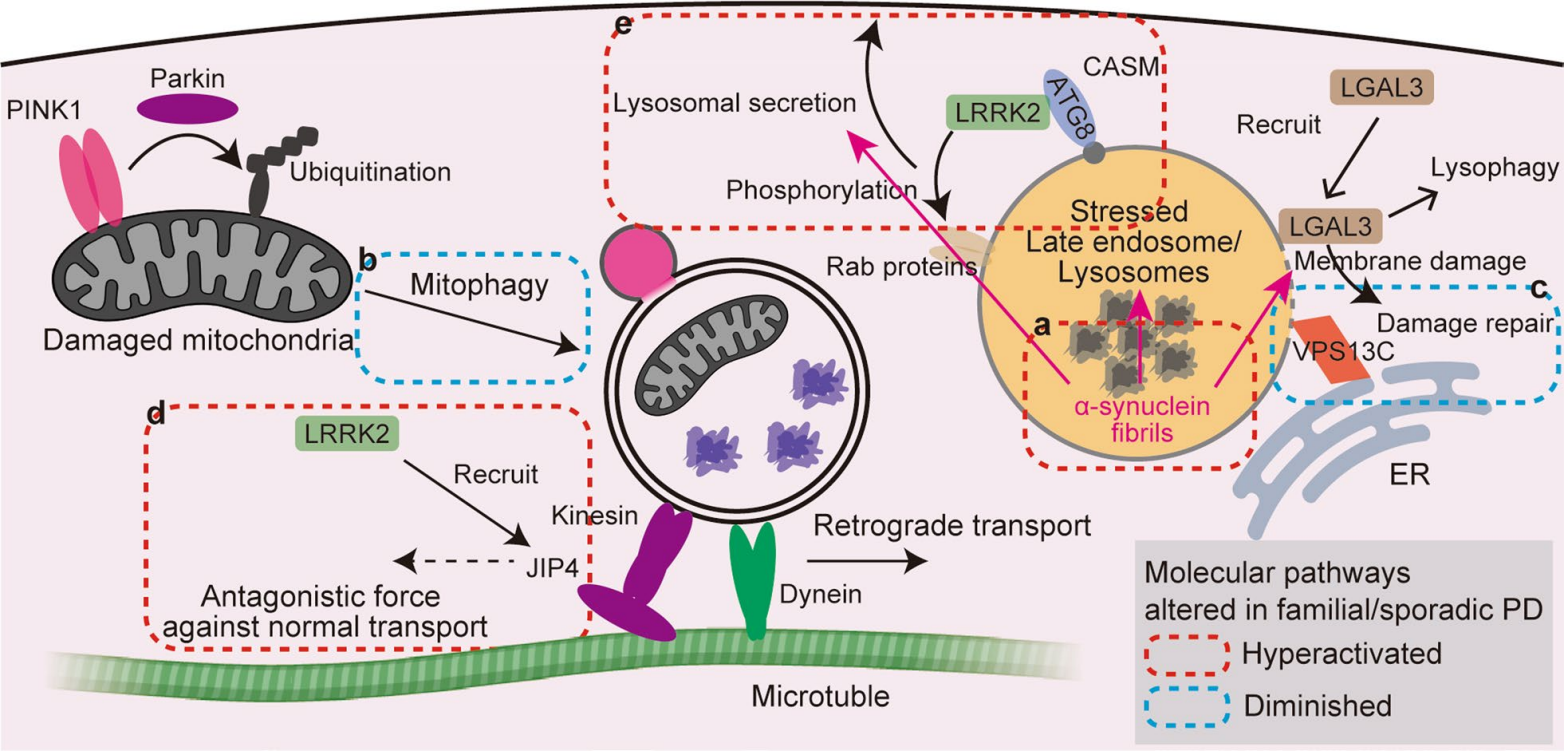
Dysregulated autophagy–lysosome pathways in familial and sporadic Parkinson’s disease (PD). **a** α-Synuclein fibrils as a source of lysosomal stress. α-Synuclein fibrils cause lysosomal stress including lysosomal membrane damage. Such stress may trigger lysophagy and the LRRK2-mediated lysosome stress responsive pathway that promotes lysosomal secretion, potentially facilitating extracellular release and propagation of pathogenic α-synuclein species. **b** Parkin/PINK1-mediated mitophagy. Upon mitochondrial damage, PINK1 accumulates on the outer mitochondrial membrane and recruits Parkin, leading to ubiquitination of mitochondrial proteins and selective clearance via mitophagy. This pathway is impaired in *Parkin*- or *PINK1*-associated PD. **c** Lysosomal membrane repair via VPS13C. VPS13C localizes at ER–lysosome contact sites and facilitates lipid transfer, contributing to lysosomal membrane repair. Loss-of-function mutations in VPS13C compromise this protective mechanism. **d** LRRK2-mediated disruption of axonal autophagosome transport. Hyperactive LRRK2 kinase phosphorylates Rab GTPases and recruits JIP4, which in turn engages kinesin motors. This may generate an antagonistic force opposing dynein-driven retrograde transport of autophagosomes, thereby impairing their delivery to the perinuclear lysosome-rich region. **e** CASM and lysosomal exocytosis under lysosomal stress. Under lysosomal overload conditions, ATG8 is conjugated to the lysosomal membrane through CASM. This process facilitates recruitment of LRRK2 and activation of lysosomal exocytosis. The pathogenic *LRRK2* mutations may potentiate this pathway

However, the extent to which the ALP pathways function effectively in vivo remains unclear. Notably, even with the depletion of soluble α-synuclein, pre-existing inclusions were found to persist, indicating that aggregated α-synuclein may evade clearance once inclusion formation has occurred [99]. In addition to their limited efficacy, recent studies suggest that autophagic pathways may, under certain conditions, be paradoxically hijacked by α-synuclein aggregates themselves to evade autophagic clearance. For instance, although α-synuclein fibrils can bind to LC3B, it has been proposed that such binding may saturate the substrate-recognition interface of LC3B, thereby interfering with the degradation of other autophagic substrates [14]. Consistent with this mechanism, previous studies reported that excess α-synuclein impairs the autophagic activity [13]. Conversely, one study suggested that excess α-synuclein can activate autophagic pathways, and that elevated mitochondrial autophagy may contribute to neuronal death by depleting mitochondria and inducing bioenergetic deficits [105]. These studies employed supraphysiological α-synuclein overexpression in cultured cells and thus may not capture the physiological settings. Nonetheless, they highlight that aberrant α-synuclein can dysregulate autophagy in either direction, enhancing or suppressing it, and importantly, that both insufficient and excessive autophagic responses can be detrimental. Furthermore, lysosomal membrane damage induced by α-synuclein aggregates has been shown to trigger lysosomal exocytosis in an ATG-dependent manner, raising the possibility that the autophagy-related mechanisms may, under certain conditions, facilitate rather than suppress the propagation of toxic α-synuclein species (Fig. 2a) [12]. In addition to damage-induced release, lysosomal overload stress alone has been reported to activate the LRRK2–Rab10 signaling axis, resulting in the extracellular discharge of lysosomal cargo (Fig. 2a) [11]. This raises the possibility that toxic α-synuclein species may also be secreted via this pathway in vivo [11].

Taken together, the relationship between toxic α-synuclein species and the autophagy machinery is highly complex, involving both protective and potentially pathogenic interactions. Further studies are needed to dissect these bidirectional mechanisms in physiologically relevant models. In particular, the development of experimental systems that faithfully recapitulate the structural and pathological features of α-synuclein fibrils in the human PD brain will be critical for advancing our understanding.

### *PRKN/PINK1*

Mutations in *PRKN* (encoding Parkin) and *PINK1* have been identified as causes for PD with autosomal recessive inheritance [71, 73]. Patients with *PRKN/PINK1*-associated PD typically present with early onset and slow progression [71, 73]. At the cellular level, Parkin and PINK1 act in concert to regulate mitophagy (Fig. 2b) [106–108]. PINK1 accumulates on the outer mitochondrial membrane and activates the E3 ubiquitin ligase Parkin, which ubiquitinates outer membrane proteins and recruits autophagy receptors such as NDP52 and OPTN to initiate mitophagy (Fig. 2b) [106–108]. This pathway provides the basis for the hypothesis that the pathogenesis of *PRKN/PINK1*-associated PD is driven by defective quality control of mitochondria. However, *Prkn-* or *Pink1*-KO mice do not develop dopaminergic neurodegeneration or motor symptoms under basal conditions, with only subtle or late-onset phenotypes reported in some cohorts at advanced ages [109–111]. Only when combined with a mitochondrial DNA stress model do these mice exhibit selective neuron loss and mitochondrial abnormalities [112]. While these findings support their role in mitochondrial quality control, it remains uncertain whether this protection is mediated primarily through mitophagy, as both proteins have also been implicated in limiting mitochondrial fusion, pointing to their broader roles in mitochondrial homeostasis [113, 114]. Notably, in a rhesus monkey model, *PINK1* deletion was associated with marked neurodegeneration in multiple brain regions, yet ultrastructural analysis reported that mitochondrial morphology in degenerating neurons was indistinguishable from that in wild-type neurons [115]. Together with the species-dependent expression and phenotypic divergence across model systems, these findings underscore that the physiological roles of PINK1/Parkin in the mammalian brain and the precise mechanisms linking their dysfunction to neurodegeneration remain incompletely defined.

Despite these discrepancies, impaired mitochondrial quality control remains a compelling disease framework for the pathogenesis of *PRKN/PINK1*-associated PD. However, a notable pathological feature in these patients, the lack of Lewy body formation, clearly distinguishes them from sPD [70, 72]. In light of these observations, caution is warranted when extrapolating findings from Parkin/PINK1 models to the broader population of sPD.

### *VPS13C*

Mutations in *VPS13C* have been identified as a genetic cause of early-onset PD, with autosomal recessive inheritance suggesting a loss-of-function mechanism [89]. Patients carrying *VPS13C* mutations typically exhibit more rapid disease progression compared to those with sPD, yet they share common pathological features such as the presence of Lewy bodies [89].

*VPS13C* is one of the four VPS13 mammalian homologs, all of which have been linked to distinct human neurological disorders, highlighting the critical roles of this protein family in CNS function [116, 117]. Each homolog appears to have a distinct localization pattern, and *VPS13C*, in particular, is found at the interface between the ER and lysosomes (Fig. 2c) [116]. Structurally, VPS13 family proteins share sequence homology with ATG2, a lipid transport protein essential for autophagosome expansion, and *VPS13C* is therefore thought to function similarly in lipid transfer [20, 116]. Indeed, Vps13 contributes to autophagosome elongation in yeast, in a manner similar to Atg2 [118]. Despite the autophagy-related function of Vps13 observed in yeast, recent studies in mammalian systems suggest that VPS13C plays a distinct role in maintaining lysosomal functional homeostasis. Upon lysosomal membrane damage, VPS13C is rapidly recruited to lysosomes earlier than LRRK2 and contributes to the maintenance of lysosomal membrane integrity, possibly via lipid transfer (Fig. 2c) [119]. In iPSC-derived dopaminergic neurons, deletion of *VPS13C* results in a range of lysosomal abnormalities, including impaired acidification, altered morphology and distribution [120].

Of particular interest is that VPS13C and LRRK2 appear to act within the same lysosomal stress response pathway, although the precise functional relationship between the two remains incompletely understood. The fact that *VPS13C*-associated PD exhibits Lewy body pathology similar to sPD, and that *LRRK2*-associated PD (LRRK2 PD) shares many clinical features with sPD [76], suggests that this lysosomal stress response pathway may play a central role in the pathogenesis of sPD. Therefore, VPS13C represents a valuable molecular clue for understanding how dysfunction of the autophagy–lysosome system contributes to the disease development. However, the lack of overt neurological symptoms in *VPS13C* KO mice highlights the current limitations of available animal models and underscores the need for more accurate systems that better recapitulate the human disease phenotype [121].

### *LRRK2*

Mutations in *LRRK2* represent the most common genetic cause of familial PD [2, 77, 79] and have also been identified as a risk factor for sPD through GWAS [4, 75]. Clinically, *LRRK2* PD closely resembles sPD in terms of age of onset and symptomatology [76], though it generally exhibits a slower disease progression [122]. Although approximately 70%–80% of *LRRK2* PD patients exhibit Lewy pathology similar to sPD, a subset of cases instead displays tau or TDP-43 accumulation, suggesting that *LRRK2* PD may involve partially distinct pathogenic processes from those of typical sPD [72, 78]. *LRRK2* encodes a large multidomain protein with kinase and GTPase activities, and pathogenic mutations generally enhance its kinase activity [123]. Given the autosomal-dominant inheritance and the lack of disease association with *LRRK2* loss-of-function variants [77, 79, 124], a gain-of-kinase function mechanism underlies disease pathogenesis. Nevertheless, the incomplete penetrance of *LRRK2* mutations suggests that additional factors may be necessary for disease onset [125].

Increased LRRK2 kinase activity has shown variable effects on the autophagic flux in cultured cells, with both enhancing and inhibitory outcomes reported [126–128]. In neurons, pathogenic LRRK2 enhances recruitment of the scaffolding protein JIP4 to autophagosomes, thereby impeding their retrograde transport and resulting in delayed autophagosome maturation and degradation (Fig. 2d) [126]. These findings suggest that the effects of LRRK2 on autophagy are mediated not by direct regulation of core ATG proteins, but rather through indirect mechanisms that are context- and cell type-dependent.

More recent studies have highlighted the role of LRRK2 in lysosomal homeostasis. *Lrrk2*-KO mice develop enlarged lysosomes and exhibit reduced lysosomal degradation capacity in lung and kidney, demonstrating a critical role for LRRK2 in maintaining lysosomal function [129, 130]. Moreover, enhanced LRRK2 kinase activity has been suggested to suppress transcription factors microphthalmia/transcription factor E, master regulators of lysosomal biogenesis and function, potentially reducing lysosomal degradative capacity [131]. Under lysosomal overload stress, LRRK2 is recruited to lysosomal membranes where it phosphorylates Rab GTPases, modulating lysosomal exocytosis [132]. Intriguingly, this recruitment appears to be mediated by CASM (Fig. 2e) [133, 134]. Because lysosomal exocytosis ultimately requires membrane fusion, the identity of the SNARE machinery operating downstream of LRRK2 recruitment remains to be defined. Notably, LRRK2 has been reported to interact with the v-SNAREs, vesicle-associated membrane protein (VAMP) 4 and VAMP7 [135], and VAMP7 also functions in the autophagosome–lysosome fusion [5]. These observations raise the possibility that LRRK2–SNARE interactions may influence whether stressed lysosomes are routed toward exocytosis versus fusion with autophagic/endo-lysosomal compartments for degradation. However, the extent to which this function contributes to LRRK2-mediated neurodegeneration remains unclear. One possible pathological mechanism is that disease *LRRK2* variants facilitate the exocytosis of aggregated α-synuclein internalized into microglial lysosomes under stress through this process, thereby contributing to intercellular spread of pathology (Fig. 2e) [11]. Notably, loss of LRRK2 causes age-dependent α-synuclein accumulation and aggregation in the rodent kidney [130], and the kidney has recently been implicated both in the clearance of circulating α-synuclein and in the kidney-to-brain pathological propagation of α-synuclein under renal failure [136]. This raises a possibility that α-synuclein spread from peripheral organ, such as kidney, may also be relevant to *LRRK2* PD. Yet, the observation that *LRRK2* PD progresses more slowly than sPD suggests that these mechanisms cannot account for the full disease phenotype.

Beyond its role in lysosomal regulation, as a multifunctional kinase, LRRK2 has been implicated in the suppression of primary ciliogenesis [137–139]. Increased LRRK2 kinase activity causes primary cilia deficiency, resulting in the disruption of Sonic hedgehog signaling and a subsequent decrease in neurotrophic factors produced by striatal cholinergic interneurons to support dopaminergic neurons [137, 139]. The primary cilia deficiency can be rescued by genetic deletion of *Rab12*, which may offer a potential therapeutic target for *LRRK2* PD [138]. Of note, autophagy and primary ciliogenesis are bidirectionally regulated [140, 141]. Thus, LRRK2-mediated ciliogenesis defect may influence autophagy regulation, and conversely, impaired autophagy caused by aberrant LRRK2 activity may contribute to ciliary dysfunction, highlighting a potential reciprocal link between these pathways in *LRRK2* PD. In addition, LRRK2 is abundantly expressed in human immune cells including microglia and has been implicated in the regulation of immune responses [142, 143]. Given the highly degradative nature of these cells, dysregulation of lysosomal homeostasis may be particularly relevant to microglial biology in *LRRK2* PD.

Given the clinical similarity of *LRRK2* PD to sPD and the presence of Lewy pathology in the majority of *LRRK2* PD cases, understanding the pathophysiology of *LRRK2* PD may offer insights into the pathogenic mechanism of sPD.

### *GBA1*

Mutations in *GBA1* were first identified as risk factors for PD in Ashkenazi Jewish populations and subsequently validated in a larger multicenter study [144, 145]. *GBA1* mutation carriers develop PD with Lewy body pathology similar to sPD [72, 146], but tend to present with an earlier age of onset and exhibit more rapid disease progression than sPD [147, 148]. These mutations reduce the activity of β-glucocerebrosidase (GCase), and greater enzymatic deficiency correlates with higher disease risk and faster progression, supporting a potential causal relationship between GCase deficiency and disease pathogenesis [149, 150].

Although GCase deficiency represents a defect in a single lysosomal enzyme, it has been shown to impair the overall lysosomal function, as evidenced by p62 accumulation, elevated lysosomal pH, and decreased cathepsin activity in both human dopaminergic neurons and *Gba1*-KO mice [151–153]. One potential mechanism involves the accumulation of glucosylceramide (GlcCer), a substrate of GCase, and its derivative glucosylsphingosine, which can activate the mTORC1 (mechanistic target of rapamycin complex 1) signaling, thereby suppressing lysosomal biogenesis and autophagic flux [154]. In addition, misfolded mutant GCase can mislocalize and bind to LAMP2A, blocking CMA [155]. GlcCer has also been shown to promote α-synuclein fibrillization, and reciprocally, α-synuclein can inhibit GCase activity in vitro [156]. Together with the observation of reduced GCase activity in sPD [157], these findings indicate that GCase-related α-synuclein pathogenesis may extend beyond *GBA1* mutation carriers.

### sPD

Direct assessment of autophagy activity in the human brain is technically not feasible, making it difficult to establish a causal link between autophagy dysfunction and sPD. Nevertheless, several pathological and molecular findings support the potential involvement of autophagy. GWAS has identified several ALP-related loci as risk factors for sPD, supporting the relevance of ALP dysfunction beyond monogenic PD [4]. Lewy bodies contain autophagy markers such as LC3, p62, and NBR1, and autophagosome-like structures accumulate in neurons in sPD brains, suggesting that the autophagy machinery is recruited to α-synuclein aggregates [158–160]. Moreover, decreased nuclear localization of transcription factor EB (TFEB), a master regulator of lysosomal biogenesis, has been reported in dopaminergic neurons from sPD patients, possibly leading to reduced autophagy–lysosomal activity [161]. An epigenomic study has identified hypermethylation of promoters of ALP-related genes in sPD brains, even prior to the widespread distribution of Lewy pathology [162]. These epigenetic changes are likely to contribute to the reduced transcription of ALP-related genes, suggesting early disruption of the autophagy–lysosomal system. While PD research has largely focused on proteostasis, a recent multi-omic evidence also points to prominent lipid dysregulation in the PD brain, and α-synuclein aggregation itself can be influenced by lipid composition [163]. In parallel, lipid-droplet-accumulating microglia have been described in aged mouse and human brains and exhibit impaired phagocytosis together with heightened oxidative and proinflammatory features [164]. Because lipid droplets can be degraded through lysosome-dependent pathways, including lipophagy, impaired lysosomal lipid handling may provide a mechanistic link between ALP function and disrupted lipid homeostasis in PD brains, with glial cells being a plausible site of vulnerability given their central roles in CNS lipid processing. Given that aging is the strong risk factor for PD onset and progression [54, 55], age-associated ALP alterations in glia could plausibly contribute to PD pathophysiology through disrupted lipid metabolism, even though the impact of aging on the autophagic flux is highly context- and cell-type-specific.

However, several issues complicate the interpretation of these findings. Since α-synuclein aggregates are ubiquitinated [158, 160], they may passively recruit proteins with ubiquitin-binding domains, such as p62 and NBR1, regardless of their active involvement in autophagic degradation. Therefore, their accumulation may simply reflect non-specific sequestration rather than functional engagement. In addition, many ALP-related transcriptional changes observed in sPD brains are also seen during normal aging, raising concerns about disease specificity [162, 165]. Finally, it remains unclear whether the transcriptional or epigenetic alterations of ALP-related genes observed in sPD represent an initiating event or a secondary change, and whether they translate into measurable impairment of autophagic flux in vivo. Nonetheless, converging evidence suggests that ALP dysfunction does occur in sPD. However, whether such a change in ALP represents a cause or a consequence of disease progression remains to be clarified. Notably, disrupted lysosomal homeostasis has been implicated in *VPS13C* and *LRRK2* PD, both of which share clinicopathological features with sPD. Together, these observations highlight the importance of thorough investigation of the maintenance of lysosomal homeostasis, particularly lysosomal repair mechanisms, in the pathogenesis of PD.

## Clinical translation of ALP in PD

### Biomarker development related to ALP

Multiple studies have explored biofluid biomarkers of PD and implicated lysosomal proteins. However, these efforts have been hindered by the small cohort sizes, yielding inconsistent or even contradictory findings. For instance, while one report showed decreased autophagy proteins, such as LC3 and p62, in the cerebrospinal fluid (CSF) of sPD patients [166], other studies failed to replicate such changes [167].

Lysosomal proteins have also been explored as candidate biomarkers. Among lysosomal enzymes, reduced GCase activity in biofluids has been the most consistent finding in *GBA1*-associated PD [168, 169]. In contrast, reports regarding GCase activity in sPD have been more variable, with some studies reporting reduced activity while others showing no significant change [169, 170]. This variability extends to other lysosomal enzymes in biofluids of sPD. For example, α-galactosidase activity is decreased in the serum of sPD patients and inversely correlates with motor and cognitive symptoms [171], whereas Cathepsin D activity is reduced in the CSF, in contrast to Cathepsin E, which is paradoxically elevated [170, 172]. Additionally, CSF levels of prosaposin, a lysosomal co-factor, may correlate with motor symptom severity, although they were reportedly unchanged between PD and control groups [173]. Regarding lysosomal membrane proteins, studies have reported inconsistent CSF levels of LAMP1 and LAMP2 in PD, which may limit their diagnostic utility [166, 167, 174]. However, LAMP2, which is a critical component of CMA, has been associated with subsequent clinical deterioration, suggesting its potential as a prognostic marker [174].

Despite the emerging evidence, the development of ALP-related biomarkers remains at an early stage. For instance, even GCase activity, the most consistently reported to be reduced in *GBA1*-associated PD, failed to show a clear separation between patients and healthy controls [168]. Considering the even greater variability and inconsistency of the ALP protein levels in biofluid across patients and across studies, their values as a diagnostic marker may be modest. In addition to biofluid, several studies have assessed ALP function in peripheral blood mononuclear cells (PBMCs) from sPD patients. For example, ex vivo proteolysis assays in cultured PBMCs, combined with lysosomal inhibition, were used to quantify the fraction of cellular protein turnover attributable to lysosome-/autophagy-dependent degradation, which was reduced in PD compared with controls [175]. More recently, PBMC lysosomal hydrolase activity/protein levels and TFEB abundance/phosphorylation were quantified, showing increased TFEB expression but limited activation and evidence of altered peripheral lysosomal enzyme dynamics in PD [176]. In line with these functional readouts, one study analyzed PBMCs from sPD patients and reported differential expression of several ALP-related genes, including *BECN1*, *ATG5*, and *HSC70* [177]. Of note, whether such peripheral changes reflect brain ALP activity remains unclear, partly because the quantitative assessment of autophagy flux in vivo in the human CNS is currently not feasible.

A key limitation in interpreting these biomarker findings is the incomplete understanding of how intracellular ALP dysfunction within the CNS leads to alterations in protein composition in biofluids. While intracellular consequences of ALP impairment have been extensively studied, much less is known about how such dysfunction alters the profile of secreted proteins. Moreover, because there is currently no established method to directly assess autophagy activity within target tissues (e.g., the human brain) in vivo, it remains difficult to correlate biofluid changes with the degree of ALP dysfunction in target tissues. To advance the field, it will be critical to characterize the full secretome of CNS-resident cells under ALP dysfunction and to develop techniques for assessing autophagy activity in live human tissues. These complementary approaches will provide a mechanistic basis for identifying ALP-related biomarkers that reliably reflect disease-relevant processes.

Given the possible heterogeneity of pathophysiology in PD, biomarker discovery efforts may benefit from stratifying patients into etiologically relatively homogeneous subgroups, such as genetically defined forms of PD, and conducting subgroup-specific analyses. Recent proteomic studies in genetically defined *LRRK2* cohorts suggest that lysosomal proteins, such as Cathepsins, are elevated in biofluids of pathogenic *LRRK2* mutation carriers, as reported in urine and CSF datasets [178–180]. Moreover, an integrated biofluid analysis across the prodromal-to-manifest spectrum indicates that several of these lysosomal proteins change in a stage-dependent manner, supporting the possibility that they function as progression-linked markers rather than static trait markers [180]. In this context, glycoprotein non-metastatic melanoma protein B (GPNMB) represents another important candidate biomarker [180, 181]. Its secretion has been reported to be regulated by LRRK2 and to increase in response to lysosomal stress, and elevated GPNMB levels in CSF have been observed in *LRRK2* mutation carriers [180, 181]. Notably, the elevation of these proteins in biofluids is consistent with reports that LRRK2 activity can mediate lysosomal protein secretion [132], providing a concrete example in which a mechanistically anchored cellular function may translate into biomarkers that also track disease-relevant processes. Such biomarkers may also serve as pharmacodynamic readouts for target engagement in therapeutic testing. Similarly, VGF is a promising biomarker candidate whose secretion is regulated by LRRK2 through its interaction with VAMP4/7, and this process is impaired by pathogenic *LRRK2* mutations [135]. Consistent with this mechanistic link, urinary proteomics has shown that VGF is decreased in PD and is among the most discriminating features for classifying both *LRRK2* mutation status and disease manifestation among *LRRK2* G2019S carriers [179]. In CSF proteomics, VGF has been identified as a PD-associated feature that contributes to distinguishing PD patients from controls [178, 182]. This example illustrates that biomarkers anchored to the molecular functions of PD risk genes, such as LRRK2, may also have utility beyond genetically defined cohorts such as a subset of sPD.

Since ALP dysfunction appears to be a common pathway of PD pathogenesis as discussed in the previous section, ALP-related biomarkers may serve as indicators of disease state or prognosis beyond diagnosis as described above. This represents an area of unmet need, given that highly sensitive diagnostic tools such as seed amplification assay are already available [183], whereas reliable biomarkers that capture disease state or progression remain largely lacking.

### Therapeutic development targeting ALP

To date, no disease-modifying therapies have been established for PD [1]. Although it remains unclear whether ALP dysfunction is a primary driver of PD in humans, activation of this pathway has been shown to ameliorate pathological features in various PD model systems [3, 184]. Furthermore, the findings from familial PD strongly suggest that maintaining lysosomal homeostasis is critical for neuroprotection. These findings provide a rationale for targeting the ALP as a disease-modifying strategy.

The most straightforward therapeutic approach is arguably in the context of familial PD, where genetic causes are well defined. One example is ambroxol, a small molecular chaperone that enhances the folding and lysosomal trafficking of GCase. In an open-label phase II clinical study, oral administration of ambroxol led to measurable CSF penetration, increased GCase protein levels, and improvement in motor symptoms even in patients without *GBA1* mutations [185]. Currently, a large placebo-controlled trial is underway to evaluate its clinical efficacy in PD. Another genetically informed therapeutic strategy involves inhibition of LRRK2 kinase activity. Since aberrant LRRK2 kinase activity is also implicated in sPD, several LRRK2 kinase inhibitors have advanced into clinical trials [186].

Beyond genetically defined subgroups, efforts have also focused on broadly targeting the ALP using pharmacological agents. These include mTOR inhibitors such as rapamycin and trehalose, AMPK (AMP-activated protein kinase) activators exemplified by metformin, and TFEB-activating compounds including lithium and curcumin derivatives [184]. Metformin and lithium, in particular, have been widely used in clinical practice for other indications and are generally considered to be relatively safe. However, despite their presumed safety, clinical evidence supporting their efficacy in PD is clearly lacking. Moreover, because many of these compounds exert broad and pleiotropic effects, therapies that selectively target the ALP are currently not available. Although not directly targeting the ALP, Abelson tyrosine kinase (c-Abl) inhibitors have been proposed as a potential therapeutic candidate. c-Abl has been shown to phosphorylate α-synuclein at tyrosine 39 [187]. This modification is commonly found in aggregates in PD brains and is associated with increased aggregation capacity [187]. In addition, c-Abl phosphorylates and inactivates Parkin, thereby impairing Parkin-mediated mitophagy [188]. c-Abl inhibitors aim to reverse these effects by reducing pathological α-synuclein aggregation and restoring mitochondrial quality control. In preclinical PD models, such inhibitors have demonstrated promising efficacy [189]. Although nilotinib, a c-Abl inhibitor originally developed for leukemia, failed to show clinical benefit in PD [190], this has been partially attributed to its limited CNS penetration [189]. Newer-generation c-Abl inhibitors with improved brain permeability are currently under clinical development [189].

Altogether, some of the ALP-modulating strategies may hold certain promising features, though their therapeutic relevance in PD remains to be clarified. Emerging evidence suggests that autophagy and lysosomal pathways may function as a double-edged sword, potentially contributing not only to cellular protection but also to the secretion and propagation of pathogenic α-synuclein species [11, 12]. Moreover, the physiological roles of ALP likely differ across cell types, indicating that broad, systemic activation of this pathway may not be uniformly beneficial. These notions highlight the need for more targeted approaches and for preclinical studies that precisely dissect cell type–specific ALP functions using experimental models. Progress toward truly targeted interventions will therefore require a comprehensive, stage-resolved understanding of what substrates fail to be adequately degraded—or, conversely, are inappropriately degraded—and the specific cell types in which these defects drive pathogenesis as PD progresses from its preclinical phases to manifest disease. Such knowledge would move the field beyond a broad “autophagy enhancement” approach and toward substrate-, cell type-, and stage-matched strategies that modulate ALP in a manner aligned with the underlying pathobiology. Furthermore, antibody-based therapies targeting α-synuclein aggregates have thus far failed to demonstrate sufficient efficacy in phase 2 clinical trials [191, 192]. While it remains unclear whether these antibodies effectively engage and remove pathological aggregates, especially in the absence of established imaging methods to visualize α-synuclein species in vivo, the failure of these trials suggests that indiscriminate removal of α-synuclein aggregates may be insufficient to reverse the disease course. These findings underscore the need for better understanding of which α-synuclein strains should be targeted, and at what disease stage such interventions would be effective.

## Conclusion

Insights from the investigation of familial PD revealed that ALP dysfunction extends across the disease spectrum, including sporadic forms. Such dysfunction encompasses not only impaired degradative activity but also defective repairing of lysosomal damage to maintain their homeostasis. Yet, clear evidence that these abnormalities are the primary cause of sPD is still lacking. A major barrier is the lack of tools that measure ALP in vivo. An additional challenge is the absence of models that faithfully recapitulate human α-synuclein strains, their cell type-specific handling, and their influence at different disease stages. While many studies have focused on degradative functions of ALP, little is known about how lysosomal damage occurs, accumulates, and is repaired over the lifespan of an organism—such knowledge is crucial for therapeutic development. Adding to the complexity, many *ATG* and lysosomal genes have functions beyond classical ALP, including roles in lysosomal repair and secretion, warranting careful interpretation of previous observations. Technical limitations also hinder progress: robust tools to monitor autophagy activity in living animals remain underdeveloped, making it difficult to establish a direct causal link between ALP dysfunction and neurodegeneration in humans. Characterizing biofluid protein changes following in vivo ALP perturbation could yield powerful biomarkers that both reflect disease state and confirm the role of ALP dysfunction in patients. Collectively, integrating improved models, refined readouts, and biomarker-driven approaches will be essential to translate mechanistic insights into biomarker development and effective therapeutic strategies.

## Data Availability

Not applicable.

## Abbreviations

PD: Parkinson’s disease
ALP: Autophagy-lysosome pathway
SNCA: Synuclein alpha
PRKN: Parkin RBR E3 ubiquitin protein ligase
PINK1: PTEN-induced putative kinase 1
PARK7: Parkinsonism-associated deglycase
LRRK2: Leucine-rich repeat kinase 2
VPS: Vacuolar protein sorting
GBA1: Glucosylceramidase beta 1
GWAS: Genome-wide association studies
ATG: Autophagy-related
CMA: Chaperone-mediated autophagy
HSC70: Heat shock cognate 71-kDa protein
LAMP2A: Lysosome-associate membrane protein type 2A
ULK: Unc51-like kinase
AMPK: AMP-activated protein kinase
PI3P: Phosphatidylinositol 3-phosphate
ER: Endoplasmic reticulum
LC3: Microtubule-associated protein light chain 3
SNARE: Soluble N-ethylmaleimide-sensitive factor attachment protein receptor
CASM: Conjugation of ATG8 to endolysosomal single membranes
CNS: Central nervous system
sPD: Sporadic Parkinson’s disease
UPS: Ubiquitin–proteasome system
VAMP: Vesicle-associated membrane protein
GCase: β-Glucocerebrosidase
GlcCer: Glucosylceramide
TFEB: Transcription factor EB
CSF: Cerebrospinal fluid
PBMCs: Peripheral blood mononuclear cells
GPNMB: Glycoprotein non-metastatic melanoma protein B
c-Abl: Abelson tyrosine kinase
